# Crotoxin Elicits Differential Increases in Macrophage Lipid Droplet Formation In Vitro Modulated during *Leishmania (Leishmania) amazonensis* Infection

**DOI:** 10.1021/acsomega.5c04319

**Published:** 2025-07-23

**Authors:** Leslye T. Ávila, Adan J. Galué-Parra, Lienne S. Moraes, Amanda A. P. Hage, Ana P. D. Rodrigues, Luis H. S. Farias, Chubert B. C. Sena, Sandra C. Sampaio, Edilene Oliveira da Silva

**Affiliations:** † Laboratory of Structural Biology, Federal University of Para, Institute of Biological Sciences, Belém, Pará 66075-110, Brazil; ‡ National Institute of Science and Technology in Structural Biology and Bioimaging, Rio de Janeiro, Rio de Janeiro 21941-902, Brazil; § Postgraduate Program in Biology of Infectious and Parasitic Agents, Federal University of Para Institute of Biological Sciences, Belém, Pará 66075-110, Brazil; ∥ Laboratory of Electron Microscopy, Department of Health Surveillance, Ministry of Health, Evandro Chagas Institute, Belém, Pará 66093-020, Brazil; ⊥ Laboratory of Pathophysiology, Butantan Institute, São Paulo, Brazil and Departments of Pharmacology and Immunology, Institute of Biomedical Sciences, University of São Paulo, São Paulo 05503-900, Brazil

## Abstract

*Leishmania* spp. is an obligate intracellular parasite that primarily infects macrophages. During infection, leukocytes are activated, culminating in the formation of lipid droplets (LDs), which are sites for the synthesis of eicosanoids that modulate the immune response. Crotoxin (CTX), the major toxin derived from *Crotalus durissus terrificus* (Cdt) snake venom, exhibits pro-inflammatory properties by promoting LD formation and further eicosanoid production. The aim of the present study was to analyze the effects of CTX on the formation of LDs in uninfected and *Leishmania (Leishmania) amazonensis*-infected macrophages. Cells were treated with CTX (2.4 and 4.8 μg/mL) and infected or not with parasites, before analyzing LD formation. The kinetic effect of CTX in uninfected macrophages demonstrated a time-dependent and progressive formation of LDs. CTX inhibited the accumulation of LDs during the first 12 h of infection and reduced the parasite load. Ultrastructural analysis revealed different electron densities and sizes of LDs in or near the parasitophore vacuole, indicating that LDs are used by the parasite. Increased levels of prostaglandin E2 (PGE2) also indicated the utilization of LDs by infected macrophages after CTX treatment. CTX stimulated the production of lipid-derived proinflammatory molecules by macrophages, thereby killing the parasite.

## Graphical abstract

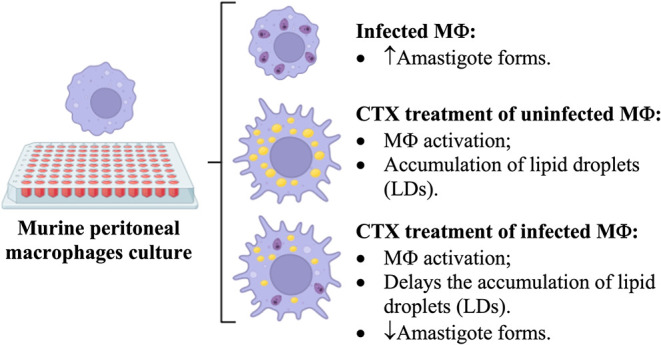

## Introduction

Infection with Leishmania spp., an obligate intracellular parasite, is responsible for a wide range of clinical manifestations, including skin and mucosal lesions. These lesions are described as tegumentary leishmaniasis and are considered a neglected disease. According to the WHO, there are 55,000 new cases of tegumentary leishmaniasis in the Americas each year.^1,2^ In Brazil, *Leishmania (Leishmania) amazonensis* is one of the major species that can inhibit the microbicidal activities of macrophages. Reduced macrophage activity causes sever skin ulcers, known as diffuse cutaneous leishmaniasis, which do not respond to conventional treatments.^3,4^

Crotoxin (CTX) is one of the major components of the venom of the *Crotalus durissus terrificus* (Cdt) snake. CTX is a heterodimeric protein composed of two subunits that are not covalently associated, the nontoxic acidic subunit (crotoxin A or CA) or crotapotin, which lacks enzymatic activity, and the basic and weakly toxic phospholipase A2 (PLA2) subunit (crotoxin B or CB).^5^ In vitro studies have demonstrated the ability of the CTX molecule to modulate immune cells.^6–8^ In a previous study, our group demonstrated the ability of CTX to stimulate the polarization of macrophages to their proinflammatory phenotype (M1) during *L. (L.) amazonensis* infection. After treatment with noncytotoxic concentrations of CTX (2.4 and 4.8 μg/mL), the macrophage phenotype is characterized by increased productions of proinflammatory cytokines, nitric oxide (NO), and reactive oxygen species (ROS), together with an elevated phagocytic index.^8^

Toxins with PLA2 activity from Cdt (CB) and other snake species are recognized as immunomodulatory molecules that are capable of inducing lipid droplet (LD) formation, prostaglandin (E2, D and J2) biosynthesis and cyclooxygenase expression.^9–11^ Snake venom and its components have been demonstrated to have antileishmanial activity and multiple immunomodulatory roles in host cells.^8,9,12^ LDs are sites of storage for intracellular neutral lipids with important cellular signaling functions.^13–15^ Their importance has increased recently, due to evidence of their role in host defense, where they represent sites of biosynthesis of prostaglandins and other microbicidal substances. However, in some cases these LDs can be induced and manipulated by pathogens as an energy source.^16^ LDs are known to be produced in high amounts in leukocytes during infectious and inflammatory processes, which correlates with the dynamic process of cell activation.^17^ LDs in leukocytes are formed from neutral lipids, especially triacylglycerides (TAG) and sterol esters (SE).^18^ During infectious diseases, LDs are the source of inflammatory mediators (eicosanoids), such as prostaglandin E2 (PGE2).^19^ These organelles have many structural proteins, such as perilipin 2 (PLN2), also called adipose differentiation protein (ADRP), which regulates lipid metabolism.^20,21^ The formation of LDs in macrophages depends on various mechanisms, including the recruitment of ADRP protein, which is stimulated by several snake venoms.^10^

LD formation is an essential process in the immune system for pathogen elimination.^22^ However, some pathogens develop strategies to use these LDs as energy sources as they are unable to synthesize essential molecules such as cholesterol.^23,24^ The search for potential therapeutic targets for combating leishmaniasis continues, as current treatments have severe side effects. Thus, the aim of the present study was to evaluate the effects of CTX on macrophages during their infection by *L. (L.) amazonensis*, focusing on the immunomodulatory effects of CTX on the accumulation of LDs.

## Materials and Methods

### Crotoxin Isolation

CTX was obtained from the lyophilized venom of Cdt, provided by Dra. Sandra Coccuzzo Sampaio from the Laboratory of Physiology, Butantan Institute, São Paulo, Brazil. The toxin was used at 2.4 and 4.8 μg/mL, from a stock concentration solution (0.945 mg/mL), stored at −20 °C and filtered through 0.22 μm membranes before each experiment.^8^

### Animals and Ethical Statements

Male BALB/c isogenic mice were used in the study at 6–8 weeks of age. Animals were kept in a temperature- and light-controlled animal room at the Institute of Biological Sciences, Federal University of Pará. All procedures were approved by the Ethical Committee for the Use of Animals (CEUA), protocol number 3297260919 (ID 001281). Experiments and animal manipulations were performed according to the guidelines of the National Council for the Control of Animal Experiments (CONCEA).

### Obtaining Murine Peritoneal Macrophages

Peritoneal macrophages were obtained from BALB/c mice sacrificed under CO_2_ atmosphere. The peritoneal cavity was washed with 5 mL of ice-cold Dulbecco’s modified Eagle’s medium (DMEM). The aspirated material was centrifuged at 4 °C, and cells were counted in a Neubauer chamber. Cells were incubated in DMEM medium at 37 °C in 5% CO_2_ for 1 h and 30 min. Nonadherent cells were washed with phosphate-buffered saline (PBS, pH 7.2), and macrophages were maintained in DMEM supplemented with 10% fetal bovine serum (FBS) (Gibco Thermo Fisher Scientific) for 24 h at 37 °C in 5% CO_2_.

### Parasites

*L. (L.) amazonensis* promastigotes (MHOM/BR/M26361) were provided by the Leishmaniasis Program of the Evandro Chagas Institute (Department of Health Surveillance, Ministry of Health, Belém, Pará, Brazil). Promastigotes were obtained in NNN medium (Novy MacNeal Nicolle) and transferred to RPMI 1640 medium supplemented with 10% bovine fetal serum FBS and maintained in a B.O.D. (Biological Oxygen Demand) chamber at 27 °C.

### Infection of Host Cells

Murine macrophages (1 × 10^6^ cells/mL) were incubated with stationary phase promastigote forms of *L. (L.) amazonensis* for 3 h at 35 °C in 5% CO_2_ at a parasite: macrophage ratio of 10:1. After incubation, cultures were washed with PBS to remove noninternalized parasites. Macrophages were treated with 2.4 or 4.8 μg/mL CTX for 1–48 h. Peritoneal macrophages were incubated without CTX (control) with DMEM supplemented with 30% FBS (positive control) and with 2.4 and 4.8 μg/mL CTX for 24 h at 37 °C in an atmosphere containing 5% CO_2_. The macrophages were then washed with PBS pH 7.2 at room temperature, fixed with 3% formaldehyde for 30 min, fixed with 4% formaldehyde for 30 min and washed with PBS. The cells were then stained with Giemsa for 1 h and washed with distilled water. The coverslips were mounted on a glass slide using Entellan as a mounting medium and analyzed using an AxioScope A1 light microscope (Zeiss).

### LDs Stained with BODIPY Dye

After CTX treatment, the accumulation of neutral and nonpolar lipids in LDs was analyzed after staining with BODIPY493/503 dye (4,4-difluoro-1,3,5,7,8-pentamethyl-4-bora-3a,4a-diaza-*s*-indacene, Molecular Probes)^25^ for 20 min at 37 °C. Cell nuclei were stained with 4′,6-diamidino-2-phenylindole (DAPI), and ProLong Gold Antifade was used to protect fluorescent dyes in macrophages cultured on coverslips. Fluorescence staining was visualized by fluorescence microscopy (AxioScope A1Microscopy, Carl Zeiss). In addition, flow cytometry was used to quantify BODIPY-stained LDs (FACSCanto II, BD Bioscience).

### Kinetics of LD Formation in Infected and Uninfected Macrophages after Treatment with CTX

*L. (L.) amazonensis*-infected and uninfected macrophages were incubated with DMEM medium supplemented with 10% FBS (control), DMEM supplemented with 30% FBS (positive control), and stimulated with 2.4 or 4.8 μg/mL CTX for 1, 3, 6, 12, 24, and 48 h, after which the cells were stained with osmium tetroxide (OsO_4_).^19^ Briefly, cells adhered to glass coverslips were fixed in 3% paraformaldehyde for 10 min, the coverslips were then rinsed in 0.1 M phosphate buffer, stained with 1.5% OsO_4_ for 30 min, rinsed in deionized H_2_O, immersed in 1.0% thiocarbohydrazide for 5 min, rinsed again in 0.1 M phosphate buffer, and stained again with 1.5% OsO_4_ for 3 min to quantify LDs within macrophages. The coverslips were rinsed with H_2_O, dried and mounted. Morphological and quantitative analysis was performed, and LDs were identified as osmiophilic black structures using AxioScope A1 microscopy (Carl Zeiss).

### Ultrastructural Analysis

Macrophages infected with *L. (L.) amazonensis*, stimulated with or without CTX, were fixed in 2.5% glutaraldehyde, 4% paraformaldehyde, 2.5% sucrose, in 0.1 M sodium cacodylate buffer, pH 7.2, for 1 h. The cells were washed three times in 0.1 M cacodylate buffer and incubated for 1 h at 4 °C in a solution containing 1% osmium tetroxide, 0.8% potassium ferrocyanide. The cells were washed three times in 0.1 M cacodylate buffer, dehydrated in graded acetone and embedded in epoxy resin and DMP30 (Sigma). Ultrathin sections were cut on an ultramicrotome (Leica EM UC6), contrasted in 5% uranyl acetate (Sigma) and lead citrate (Sigma), and viewed on a LEO 906E transmission electron microscope (Carl Zeiss).

### Measurement of PGE2 Levels

PGE2 levels were measured in the supernatants of infected macrophages (control), and infected macrophages stimulated with CTX (2.4 and 4.8 μg/mL) or LPS/INF-γ (positive control) for 48 h. PGE2 measurement was performed with the BiotrakProstaglandin E2 competitive immunoenzymatic commercial assay (GE Healthcare), according to the manufacturer’s instructions. The result was read by spectrophotometry at a wavelength of 450 nm in a microplate reader (ELx800, Biotek).

### Statistical Analysis

All biological experiments were done thrice with triplicates of each group. Statistical analyses were performed with GraphPad Prism 6.0. Multiple comparisons between groups were performed with one-way ANOVA followed by Tukey’s test, and unpaired Student *t* test was used for the comparison of two groups of data. *p* < 0.05 (*), *p* < 0.01­(**), and *p* < 0.001 (***), were considered statistically significant.

## Results

### Crotoxin Induces Morphological Changes and LD Formation in Macrophages

Morphological changes and LD formation in CTX-stimulated macrophages. For this, cells were cultured with DMEM supplemented with 10% (control) or 30% FBS (positive control for LD formation) and treated with 2.4 or 4.8 μg/mL CTX for 24 h. After Giemsa and osmium tetroxide (OsO_4_) staining (Figure 1A,B), the CTX-treated cells presented an accumulation of vacuolar LD-like structures in their cytoplasm.

**Figure 1:**
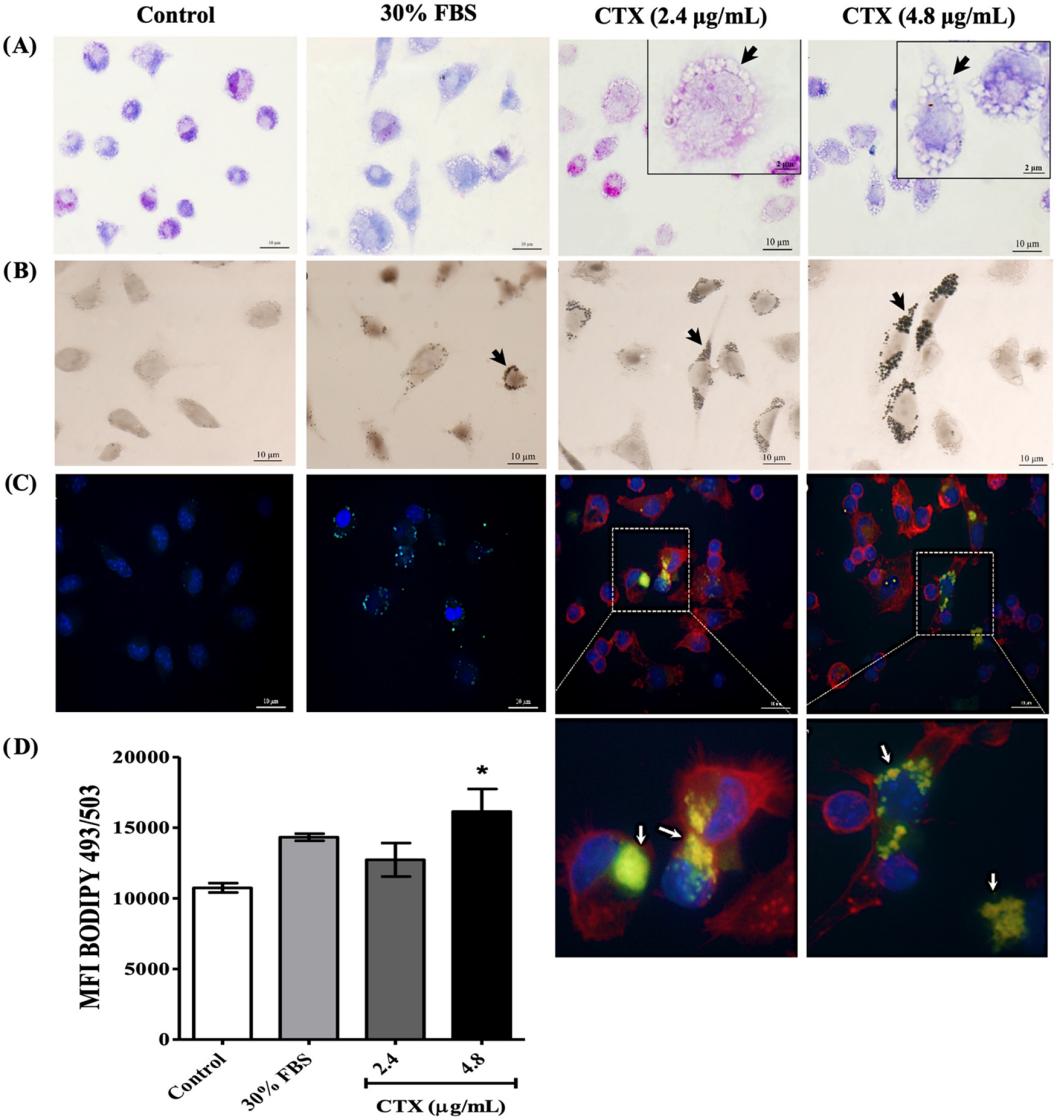
Morphological and cytochemical analysis of macrophages after CTX treatment. (A) Giemsa-stained macrophages present a high number of vacuolar structures in the cytoplasm of treated cells. (B) Osmium tetroxide and (C) BODIPY493/503 staining demonstrates the accumulation of lipid droplets in CTX-treated cells. (D) Median fluorescence intensity (MFI) of BODIPY493/503 quantified in stained macrophages; a significant increase in fluorescence was observed after treatment with 4.8 μg/mL CTX, compared to the control group. Data are shown as means and standard deviation of three independent experiments in triplicate, and analyzed by ANOVA with Tukey post test. *p < 0.05, compared with control group. Arrows: lipid droplet accumulation; DAPI (blue fluorescence); phalloidin (red fluorescence); BODIPY493/503 (yellow fluorescence); bars: 10 and 2 μm.

To confirm the formation of LDs in response to CTX stimulation, the macrophages were stained with BODIPY 493/503, a marker for neutral and nonpolar lipids (Figure 1C). Stained lipid droplets were easily visualized by fluorescence microscopy after treatment. Quantitative analysis was performed using flow cytometry (Figure 1D); results show that only the concentration of 4.8 μg/mL CTX induced a significant increase in LDs in cells.

### Kinetics of LD Formation in CTX-Treated Murine Macrophages

To demonstrate the effect of CTX on LD formation, over time, in macrophages stimulated with 2.4 or 4.8 μg/mL CTX, cells were stained with osmium tetroxide (OsO_4_) at different times during 48 h (Figure 2). LDs rapidly accumulated in a time-dependent manner only when the macrophages were treated with CTX, compared with the control group (Figure 2A). Quantification of OsO_4_-stained vesicles showed that both concentrations of CTX induced a similar increase in the number of LDs during the 48-h analysis, except at 24 h, where the cells treated with 2.4 μg/mL CTX presented a significantly lower number of LDs than the cells treated with 4.8 μg/mL CTX (Figure 2B). Another important finding was that CTX induced higher number of LDs than the positive control (30% FBS group), at all time points analyzed (Figure 2B).

**Figure 2:**
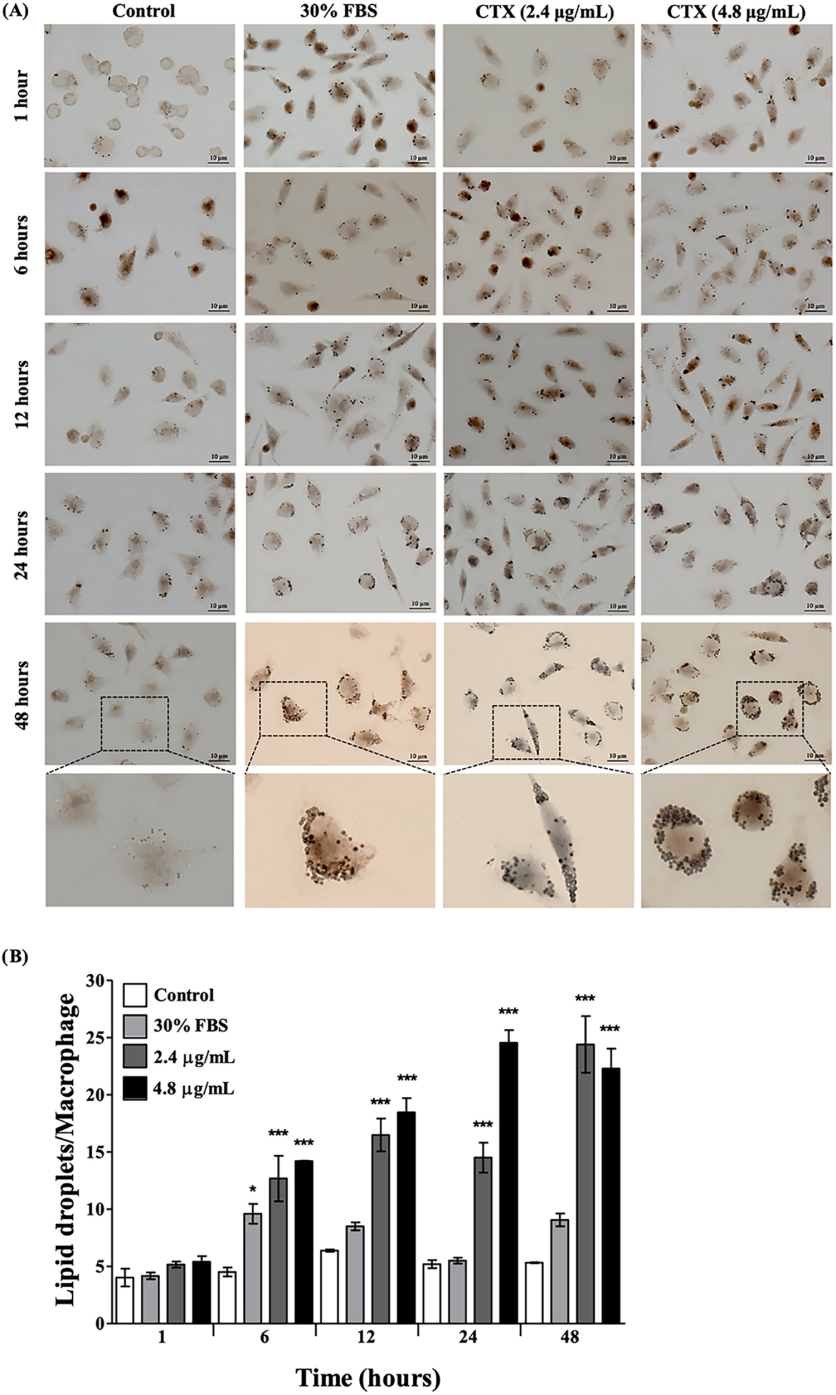
Kinetics of LD formation in macrophages. Macrophages were incubated with DMEM (control MF) or stimulated to produce LDs with FBS (30%) or stimulated with CTX (2.4 or 4.8 μg/mL) for different time periods (1–48 h). LDs were stained with OsO_4_ and visualized by light microscopy (A) and quantified (B). Note the significant increase in LDs in CTX-stimulated macrophages, compared to the unstimulated control group. Note that the formation of these organelles is time-dependent, with a significant increase at 48 h. Values represent the means ± SEM of three independent experiments with 3–5 animals. *p < 0.05, **p < 0.01, ***p < 0.001, compared to control (ANOVA, Tukey test). Bar: 10 μm. Arrows: LDs.

### Evaluation of LD Formation in CTX-Stimulated Macrophages During *L. (L.) amazonensis* Infection

In order to evaluate the infection process by *L. (L.) amazonensis* in peritoneal macrophages stimulated with CTX, images were taken at different time points of the study after OsO_4_ staining. The differentiation from promastigote to amastigote forms occurred simultaneously with the formation of LDs, and the cytoplasm of promastigotes also presented accumulation of LDs. Subsequently, as the number of LDs in the macrophage increased, the number of amastigote forms decreased, especially after 48 h of infection, as shown in Figure 3A.

**Figure 3:**
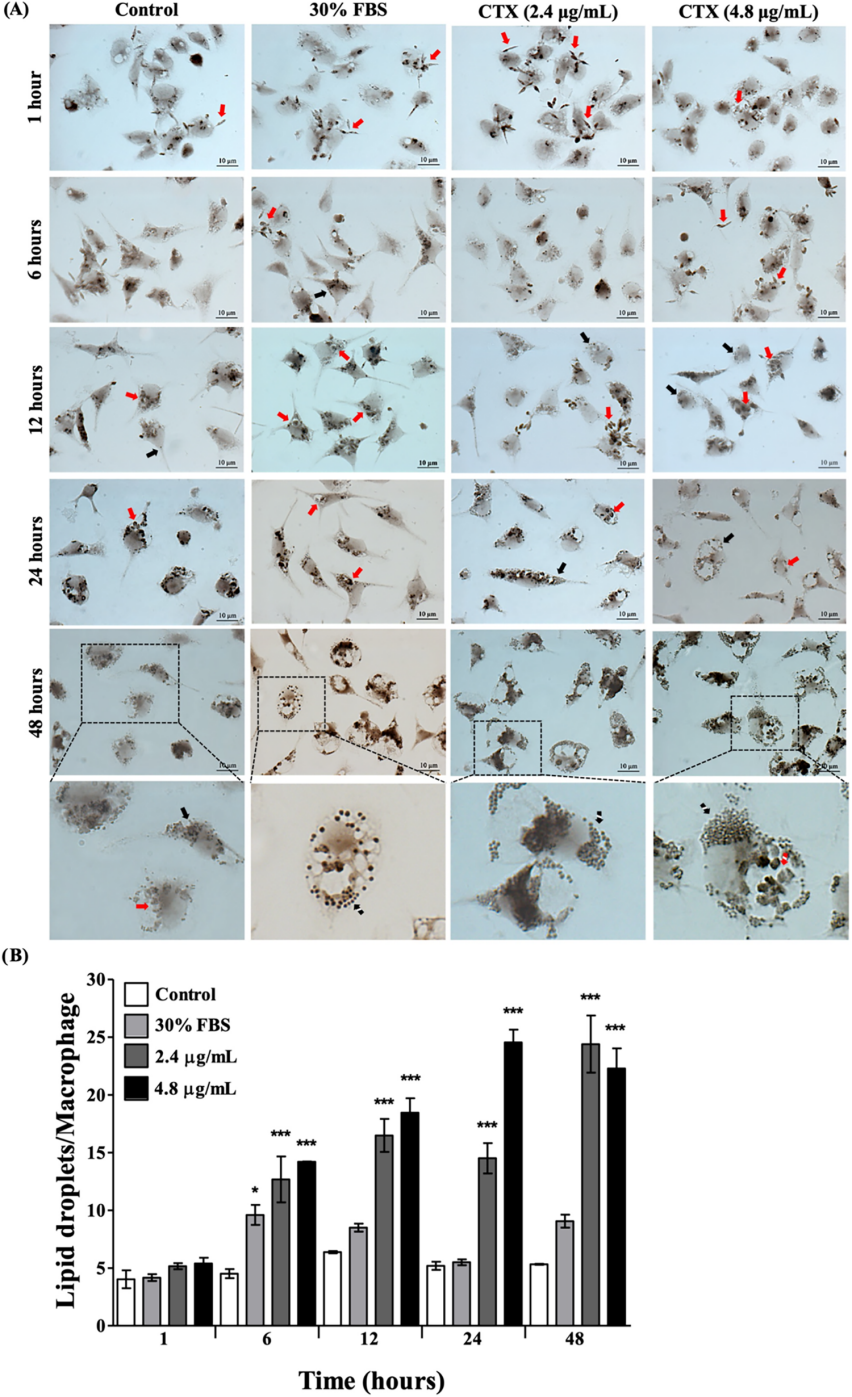
Macrophages infected with *L. (L.) amazonensis* and treated with CTX. Peritoneal macrophages cultured in DMEM with 10% FBS or 30% FBS (Mφ + L. am + FBS 30%) to induce LD formation were infected with *L. (L.) amazonensis* promastigotes for 3 h and stimulated with 2.4 or 4.8 μg/mL CTX for 48 h. Infected cells were stained with OsO_4_ at different times of infection to kinetically visualize (A) and quantify (B) LDs. Note the significant increase in LDs in infected macrophages after CTX stimulation, especially at 48 h. Parasites are indicated by red arrows and LDs by black arrows. Values represent the means ± SEM of three independent experiments with 3 to 5 animals. *p < 0.05, **p < 0.01, ***p < 0.001, compared to control (ANOVA).

The kinetic quantification of LD formation during infection with *L. (L.) amazonensis* and CTX-stimulated macrophages is shown in Figure 3B. DMEM supplemented with 30% FBS was used as a positive control to increase the formation of LDs. The biogenesis of lipid droplets formation was not linear during the initial period of infection, as observed without infection. During the first 6 h of infection, the amount of lipid droplets decreased in all groups, including the 30% FBS control group and the CTX-treated groups. However, after 12 h of infection, the number of LDs began to increase significantly. Despite this increase, LDs levels remained lower than those seen in uninfected cells until 24 h, compared with the results of Figure 2. Only after 48 h of infection did the number of LDs increase to levels similar to those observed in uninfected cells, where significant numbers of LDs were found in the 30% FBS control group and CTX-treated groups. Another important finding at 48 h was that infected macrophages also showed an increase in LDs without treatment. However, this LD formation was less than in the CTX-stimulated groups. These data suggest that *L. (L.) amazonensis* initially tries to inhibit the overproduction of LDs in macrophages during the transformation to amastigote forms.

To correlate these findings, uninfected cells (from Figure 2) and infected cells (from Figure 3) at 12 and 24 h after treatment with 30% FBS (positive control) or 2.4 and 4.8 μg/mL CTX, shows that Leishmania infection concentrates LDs in parasites and PVs after 12 h, regardless of treatment (Figure S1 of Supporting Information). However, after 24 h of treatment, the LDs began to accumulate in the cytoplasm of macrophages during CTX treatment, suggesting that CTX decreased the viability of the parasites to use the LDs by inducing the M1 proinflammatory macrophage profile to kill the intracellular parasites.

### LD Biogenesis and Amastigote Transfomation in CTX-Treated Macrophages Infected by *L. (L.) amazonensis*

To test whether Leishmania infection inhibits the overproduction of LDs, we quantified LDs in uninfected and infected macrophages after treatment with 4.8 μg/mL CTX (Figure 4A). The data clearly show that during the early hours of infection (up to 24 h), the number of LDs in macrophages after CTX treatment decreased only when *L. (L.) amazonensis* infection occurred. However, at 48 h, a significant increase in the number of LDs was observed in infected cells, compared to uninfected cells. To determine the role of CTX-induced LD formation, in relation to intracellular amastigote transformation in infected macrophages, the number of amastigotes was quantified in 100 infected macrophages after 48 h of infection. As shown in Figure 4B, both concentrations of CTX (2.4 and 4.8 μg/mL) induced a significant reduction in amastigote forms, compared to untreated CTX cells.

**Figure 4:**
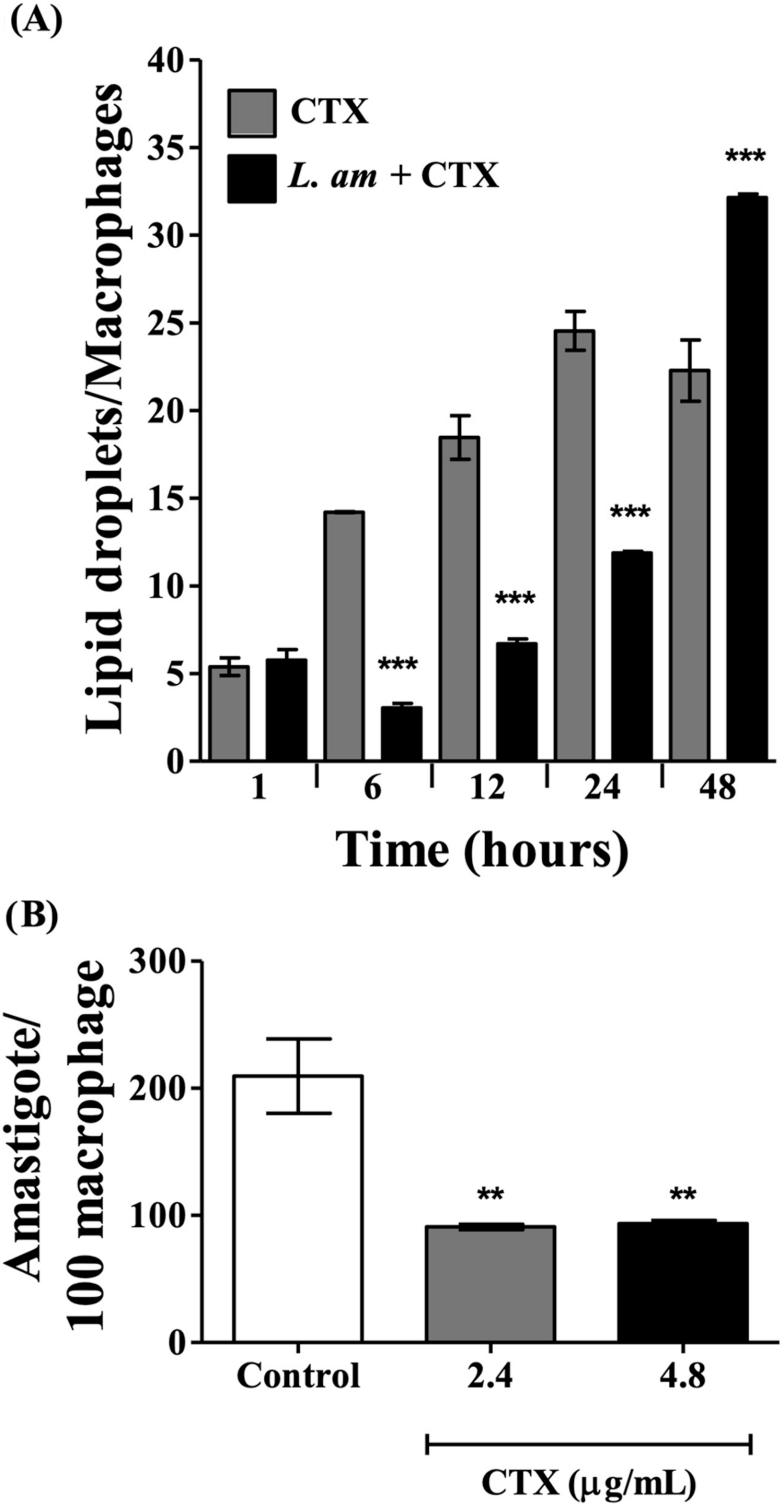
CTX-induced LD formation is modified by *L. (L.) amazonensis* infection and affects the amount of amastigote forms. (A) Infected and uninfected macrophages were stimulated with CTX. Note a significant decrease in LD formation at 6, 12, and 24 h and a significant increase in LDs in stimulated infected macrophages at 48 h. (B) Intracellular amastigotes were quantified at 48 h of infection in 100 macrophages, with and without CTX treatment (control). A statistically significant reduction in parasites was observed, independently of the CTX concentration. **p < 0.01, ***p < 0.001, compared to control (ANOVA, Tukey test).

### Ultrastructural Changes in *L. (L.) amazonensis* Infected Macrophages during CTX Treatment

We analyzed the ultrastructural characteristics of macrophages at 24 h of CTX treatment, with and without infection by *L. (L.) amazonensis*. An analysis at 24 h was performed because macrophages begin to accumulate LDs at this time during infection, facilitating the initial evaluation of the role of CTX in inducing LD formation. Figure 5A shows an infected macrophage without treatment with CTX. During CTX treatment (Figure 5B–E), LDs appear and release their contents into the parasitophorous vacuole (Figure 5B). They also appear inside (Figure 5C,D) and close to (Figure 5E) the parasitophorous vacuole. Another important finding was that the parasites have their own lipid droplets (Figure 5D,E). These data suggest that *L. (L.) amazonensis* can also use LDs during infection, either by capturing them directly from the macrophage cytoplasm or by forming them themselves.

**Figure 5:**
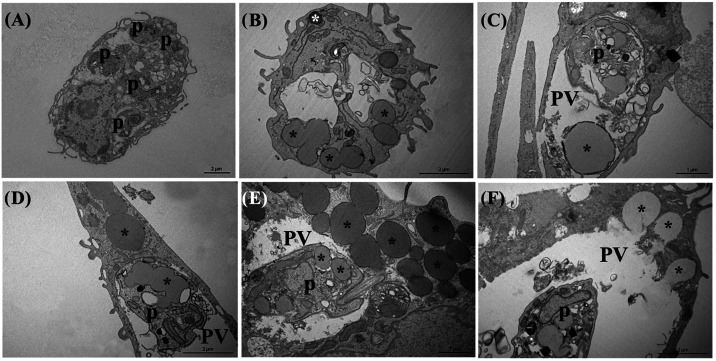
Ultrastructural analysis of infected untreated macrophages and *L. (L.) amazonensis*-infected macrophages after 24 h of treatment with 2.4 or 4.8 μg/mL CTX. (A) Untreated and infected macrophage. (B) 2.4 μg/mL CTX-treated macrophage showing the fusion of LDs (black asterisk) and also LDs with different densities in the same cell (white asterisk). (C) and (D) 2.4 μg/mL CTX-treated and infected macrophage showing LDs inside the parasitophorous vacuole (C), parasite showing its own LDs (D) and many LDs of different sizes inside the host cell. (E, F) 4.8 μg/mL CTX-treated and infected macrophage showing LDs releasing their contents into the parasitophorous vacuole. PV: parasitophorous vacuole; and p: parasite.

### Quantification of Prostaglandin E2 in the Supernatant of Infected Peritoneal Macrophage Cultures during CTX Treatment

To evaluate the effect of CTX on the pro-inflammatory response, we measured concentrations of prostaglandin E2 (PGE2) in the supernatants of *L. (L.) amazonensis*-infected macrophages after treatment with 2.4 or 4.8 μg/mL CTX for 48 h. As shown in Figure 6, the CTX-treated macrophages and positive control macrophages (LPS and INF stimulated group) released significantly higher concentrations of PGE2 than the control group (infected and not treated with CTX). These data indicate that the stimulation of LD formation by CTX may induce the production of bioactive lipids such as PGE2, which play a role in infection control by the host. This is supported by the decreased number of amastigotes after 48 h of infection and CTX treatment.

**Figure 6:**
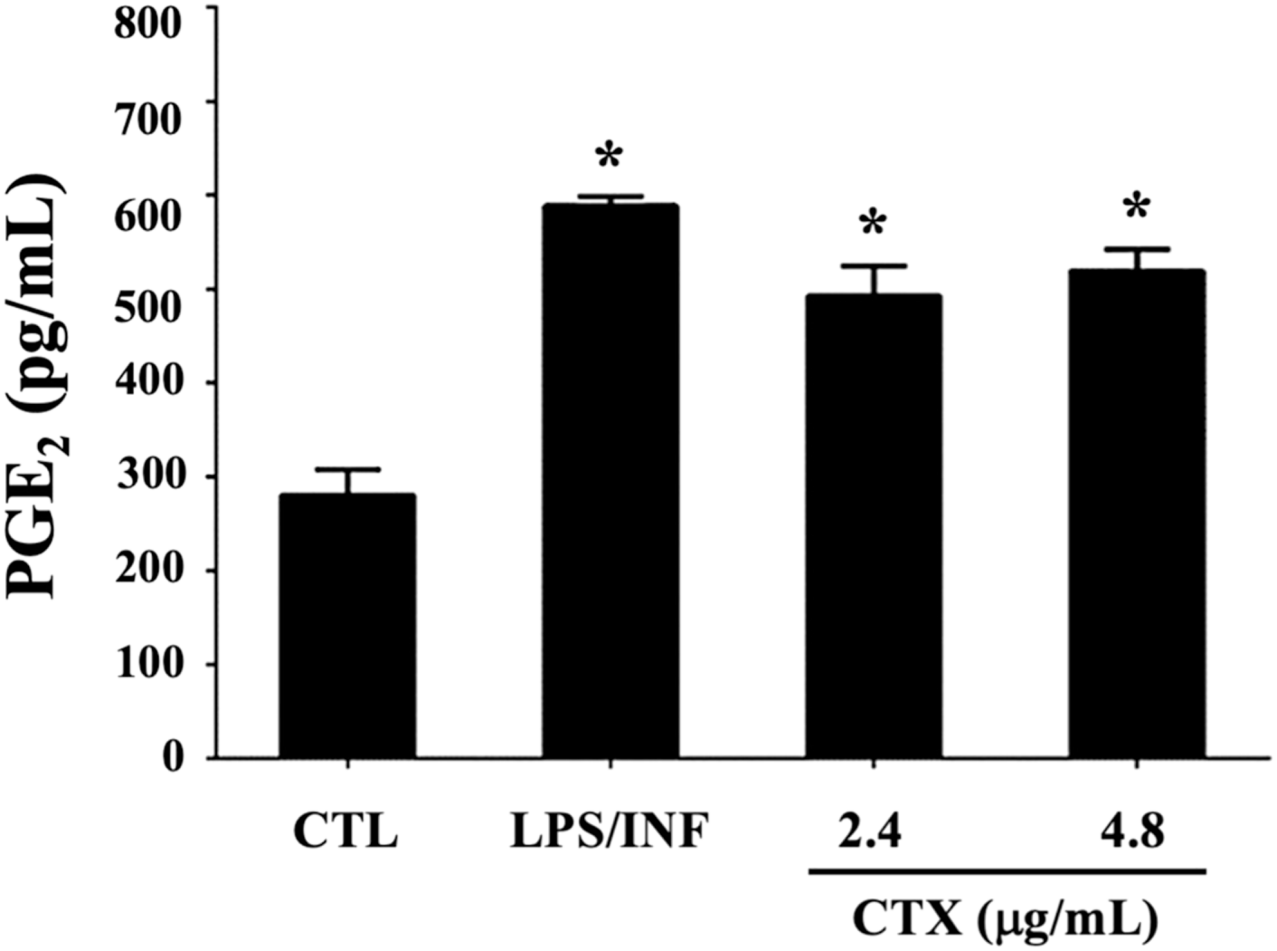
PGE2 levels during infection and in CTX-treated macrophages. *L. (L.) amazonensis*-infected macrophages were incubated with DMEM (CTL, negative control), LPS/INF- (positive control) or 2.4 or 4.8 μg/mL CTX for 48 h. Infected cells treated with CTX showed a significant increase in PGE2, compared to the control group, regardless of the concentration of CTX used. *p < 0.05 (ANOVA, Tukey test).

## Discussion

Our group previously reported that CTX, the major snake venom toxin from *C. durissus terrificus* with phospholipase A2 activity, might have therapeutic potential against leishmaniasis. We found that CTX stimulates murine macrophages toward the pro-inflammatory M1 phenotype during *L. (L.) amazonensis* infection, increases the phagocytic index of macrophages and decreases the survival of parasites, thereby improving the cellular response against cutaneous leishmaniasis.^8^

LDs are present in all cell types, including protozoan parasites and activated macrophages. In parasites, LDs provide neutral lipids and, in macrophages, they act as a source of pro-inflammatory cytokines.^26^ Importantly, fatty acids and their metabolism are a potential therapeutic target for treating leishmaniasis.^27,28^ LDs are observed inside the parasitophorous vacuole and inside the Leishmania parasite, which uses it as an alternative energy source during the infection of macrophages.^28^ Phospholipase A2 from snake venom is known to induce LDs and also synthesize pro-inflammatory lipid mediators.^7,9,29^ The present study showed that CTX enhanced the cytoplasmic accumulation of neutral lipids in uninfected macrophages (Figure 1). LDs contain neutral lipids, such as triacylglycerol and cholesterol ester,^18^ confirming the biological role of CTX in inducing the accumulation of LDs.^9,10^ Our kinetic analysis of LD accumulation showed that, after 6 h of CTX treatment, macrophages overproduced LDs, with this production persisting for at least 48 h. This LD accumulation profile was not seen for the 30% FBS positive control, which induced LD formation only after 6 h of stimulation (Figure 2). Thus, CTX can induce LD formation in macrophages in a time-dependent manner, compared to the unstimulated control group. The accumulation of LDs in macrophages may occur independently of infection and be stimulated by exogenous stimuli,^28^ as seen after treating cells with 30% FBS or CTX.

During macrophage infection by *L. (L.) amazonensis* and CTX stimulation, the formation of LDs was inhibited by the parasite during the first 12 h (Figure 3). It is well-known that Leishmania induces the accumulation of LDs during macrophage infection and recruits them to the parasitophorous vacuoles (PVs). These LDs are used not only as an alternative energy source, but also to hijack important molecules that activate the host and enable the parasite to survive during infection.^30,31^ Figure S1 confirms this biogenesis of LDs during infection, showing that the accumulation of LDs in the cytoplasm of macrophages only begins after 24 h of infection. We previously showed that CTX does not directly interfere with the activity of extracellular promastigotes, but it can overcome the immunosuppressive parasite strategy and develop M1 proinflammatory macrophages during Leishmania infection,^32^ which was confirmed by prostaglandin E2 (PGE2) production in Figure 6. This corroborates the anti-Leishmania activity of CTX. This biological activity of CTX is related to its phospholipase A_2_ (PLA_2_) activity, which is known to form LDs and inflammatory lipid mediators such as PGE_2_.^33^ The inhibitory effect of *L. (L.) amazonensis* on LD accumulation during CTX treatment is more evident when comparing uninfected and infected macrophages during 48 h (Figure 4A). *Leishmania* infection clearly decreased the induction of LD formation by CTX during the initial 24 h of infection, however at 48 h the infected CTX-treated cells accumulated 30% more LDs than uninfected CTX-treated cells. These results suggest that *L. (L.) amazonensis* is utilizing the LDs during the first 24 h of infection in the presence of CTX treatment. *Leishmania* is known to modulate the accumulation of LDs by using them as energy sources during the course of infection, helping the differentiation of promastigotes into amastigotes,^28^ as is seen in the infected cells that are not stimulated with CTX (control group, Figure 3). However, compared to the untreated infected group, the number of amastigote forms significantly decreased after 48 h of CTX treatment (Figure 4B). This suggests that the increased accumulation of LDs at that time is due to the death of the parasite died, indicating that the proinflammatory effects of CTX treatment override the use of LDs as an alternative nutritional source for the parasites during the infection.

During the infection of macrophages with *L. (L.) amazonensis*, together with CTX stimulation, LD formation was inhibited by the parasite during the first 12 h (Figure 3). The inhibitory effect of *L. (L.) amazonensis* on LD accumulation during CTX treatment can be better observed when comparing uninfected and infected macrophages over 48 h (Figure 4A). *Leishmania* infection significantly decreased CTX-induced accumulation of LDs until 24 h of infection, however, at 48 h the infected CTX-treated cells accumulated 30% more LDs than uninfected CTX-treated cells. These results suggest that *L. (L.) amazonensis* utilizes the LDs during the first 24 h of infection and CTX treatment, decreasing the accumulation of LDs up to 24 h. *Leishmania* can modulate the accumulation of LDs by using them as an energy source during the course of infection to help the cells to differentiate from promastigotes to amastigotes cells,^28^ as can be seen when infected cells were not stimulated with CTX (control group of Figure 3). However, compared with the group that was not treated with CTX, the number of amastigote forms significantly decreased after 48 h of CTX treatment (Figure 4B). This result indicates that LD accumulation is accelerated at this stage of the infection during CTX treatment, due to the death of the parasite at this stage, suggesting that the proinflammatory effect of CTX treatment on *Leishmania* infection supersedes its ability to act as an alternative nutritional source for parasites.

Ultrastructural analysis at 24 h of infection and CTX treatment showed that LDs accumulated inside the uninfected and CTX-treated macrophages (Figure 5B). However, after infection, the LDs can be seen accumulated inside the parasite, around the parasitophorous vacuoles or releasing their contents into them (Figure 5C–F). The biology of various *Leishmania* species has been linked to fatty acid metabolism. This includes modulation of metacyclogenesis, antileishmanial drug resistance, parasite infectivity, and also the development of leishmaniasis.^27^ Translocation of LDs to the parasitophorous vacuole has been observed not only in *Leishmania*, but in various intracellular protozoan parasites. This mechanism allows the parasite to obtain nutrients and produce anti-inflammatory mediators to evade the host immune response.^8,23,26,28^

The hypothesis that the proinflammatory effects of CTX treatment during Leishmania infection supersede the toxin’s ability to act as an alternative nutritional source for parasites was confirmed. This was supported by the finding that infected macrophages treated with CTX produced high levels of PGE2, as did the LPS/INFγ positive control group (Figure 6). PLA2-derived venoms from various Crotalus snake species, such as CTX, are known to induce the synthesis of inflammatory lipid mediators from LDs, such as prostaglandins (PGE2, PGD2, and TXB2), and the expression of cyclooxygenases (COXs).^9,10,29^ CTX-induced LDs are also associated with the expression and activation of kinase proteins (PKC, PI3K, MEK1/2, and JNK).^9,10^

In conclusion, our results show that CTX-induced LD formation during *L. (L.) amazonensis* infection initially provides an energy source for the parasite, as an alternative carbon source, thereby reducing the accumulation of LDs during the first 24 h of infection. However, the parasites are unable to survive the inflammatory response that is also induced by CTX. As such, this is the first study to analyze the CTX-induced biological accumulation of LDs in macrophages during *L. (L.) amazonensis* infection. CTX administration may represent an alternative strategy to treat the lesions of patients with leishmaniasis.

## Supplementary Material



## Notes

Uninfected and infected macrophages with *L. (L.) amazonensis* treated with CTX (Figure S1) (PDF)

## Abbreviations

LDs: lipid droplets
CTX: Crotoxin
Cdt: *Crotalus durissus terrificus*
PGE2: prostaglandin E2
ROS: reactive oxygen species
