# Supplementary material for: Crotoxin Elicits Differential Increases in Macrophage Lipid Droplet Formation In Vitro Modulated during Leishmania (Leishmania) amazonensis Infection
Source: ACS Omega. 2025 Jul 23;10(30):33619–28. doi: 10.1021/acsomega.5c04319 (PMC12366855; doi:10.1021/acsomega.5c04319)
Supplement: Supplementary file 1 [file ao5c04319_si_001.pdf]

Crotoxin elicits differential increases in macrophage lipid droplet formation in vitro modulated during Leishmania (Leishmania) amazonensis infection

Leslye T. Ávila [a,c], Adan J. Galué-Parra [a,b,c], Lienne S. Moraes [a], Amanda A. P. Hage [a], Ana P. D. Rodrigues [c,d], Luis H. S. Farias [a], Chubert B. C. Sena [a], Sandra C. Sampaio [e], Edilene Oliveira da Silva [a,b,c].*

[a] Laboratory of Structural Biology, Federal University of Para, Institute of Biological Sciences, Belém, Pará, Brazil;

[b] National Institute of Science and Technology in Structural Biology and Bioimaging, Rio de Janeiro, Rio de Janeiro, Brazil;

[c] Postgraduate Program in Biology of Infectious and Parasitic Agents, Federal University of Para Institute of Biological Sciences, Belém, Pará, Brazil;

[d]Laboratory of Electron Microscopy, Department of Health Surveillance, Ministry of Health, Evandro Chagas Institute, Belém, Pará, Brazil;

[e] Laboratory of Pathophysiology, Butantan Institute, São Paulo, Brazil and Departments of Pharmacology and Immunology, Institute of Biomedical Sciences, University of São Paulo, São Paulo, Brazil;

*Email: edilene@ufpa.br

KEYWORDS: Leishmania (Leishmania) amazonensis; lipid droplets; crotoxin.

Supporting information

Figure S1.

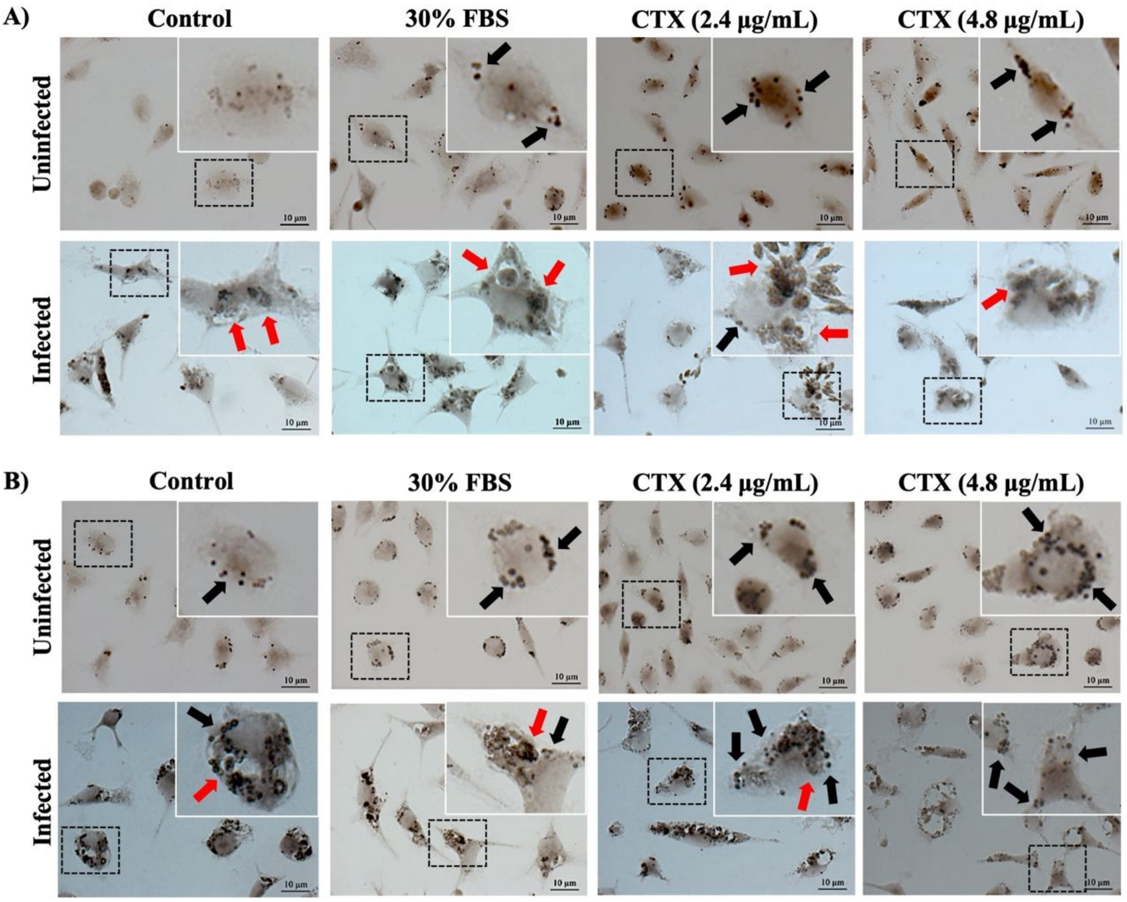

Figure S1. Uninfected and infected macrophages with *L. (L.) amazonensis* treated with CTX. Peritoneal macrophages were cultured in DMEM with either 10% or 30% FBS to induce LD formation, then infected with L. (L.) amazonensis promastigotes for three hours and stimulated with either 2.4 or 4.8 µg/mL CTX for 12 or 24 hours (panels A and B, respectively). Note that infection decreases cytoplasmic LDs after 12 hours of CTX stimulation (Figure A) and increases the accumulation of cytoplasmic LDs after 24 hours of infection. Parasites are indicated by red arrows and LDs by black arrows.
